# Development of RP-HPLC Method for Simultaneous Determination of Triclabendazole and Ivermectin in Pharmaceutical Suspension Dosage Form

**DOI:** 10.1155/jamc/5522915

**Published:** 2025-04-22

**Authors:** Taj Ur Rahman, Ajmal Zaman, Ali Bahadur, Muhammad Aurang Zeb, Wajiha Liaqat, Eman Y. Santali, Sarah Alharthi, Ruwida M. K. Omar, Saif A. Alharthy, Ashraf Ali

**Affiliations:** ^1^Department of Chemistry, Mohi-Ud-Din Islamic University, Nerian Sharif, Azad Jammu and Kashmir, Pakistan; ^2^Department of Transdisciplinary Studies, Graduate School of Convergence Science and Technology, Seoul National University, Seoul 08826, Republic of Korea; ^3^Department of Chemistry, School of Natural Sciences (SNS), National University of Science and Technology (NUST), H-12, Islamabad 46000, Pakistan; ^4^Department of Pharmaceutical Chemistry, College of Pharmacy, Taif University, Taif 21944, Saudi Arabia; ^5^Department of Chemistry, College of Science, Taif University, P.O. Box 11099, Taif 21944, Saudi Arabia; ^6^Research Center of Basic Sciences, Engineering and High Altitude, Taif University, Taif 21944, Saudi Arabia; ^7^Pharmaceutical Chemistry Department, Faculty of Pharmacy, University of Benghazi, Benghazi, Libya; ^8^Department of Medical Laboratory Sciences, Faculty of Applied Medical Sciences, King Abdulaziz University, P.O. Box 80216, Jeddah 21589, Saudi Arabia; ^9^King Fahad Medical Research Center, King Abdulaziz University, P.O. Box 80216, Jeddah 21589, Saudi Arabia; ^10^Department of Chemistry, Faculty of Physical and Applied Sciences, The University of Haripur, Haripur 22620, Pakistan; ^11^School of Chemistry & Chemical Engineering, Henan University of Technology, Zhengzhou 450001, China

**Keywords:** liquid chromatography, method development, method validation, pharmaceutical analysis

## Abstract

A reversed-phase high-performance liquid chromatography (RP-HPLC) method was developed for simultaneous determination of triclabendazole (TCB) and ivermectin (IVM) in pharmaceutical dosage form. A mobile phase consisting of acetonitrile/water (50:50 v/v) with a flow rate of 1.5 mL/min was used for chromatographic separation of the mixture of TCB and IVM. The developed method was found to be linear with the correlation coefficient (*r* = 0.999) for TCB and IVM in the presence of suspension. The limit of quantitation (LOQ), robustness, specificity, accuracy, and precision were validated for the developed method. The peak areas of five replicates of the samples were recorded, and the acceptance rate of suspension recovery was 98%. The intraday accuracies for TCB and IVM were 98.71% and 100.79%, respectively, with a relative standard deviation (RSD) of 0.87%. The limits of detection (LOD) of TCB and IVM were 0.058 mg/mL and 0.112 μg/mL, respectively, while the LOQ of TCB and IVM were 0.178 μg/mL and 0.340 μg/mL, respectively. The method's % RSD for intra- and interday precision was deemed satisfactory. The developed method could be utilized for the determination and measurement of TCB and IVM in other samples.

## 1. Introduction

In the pharmaceutical industry, it is critical to determine bulk drug materials, intermediates, contaminants, drug formulations, degradation products, and associated metabolites with great accuracy and precision [1]. However, as the patient's health is directly impacted by the quality control of pharmaceutical products, the clear identification of pharmaceuticals in pharmaceutical formulations is equally significant [2]. Chemical analysis is essential for pharmaceutical control and drug development in order to guarantee high patient safety and efficacy. For this reason, the pharmaceutical sector places a high priority on adequate and genuine techniques of quality control [3]. Very selective and innovative analytical techniques are consequently needed for the separation and purification of the very complex compounds and medication formulations that are the product of pharmaceutical research and development [4]. For this reason, suitable analytical techniques ought to be created in order to regulate the caliber of pharmaceutical analysis [5]. Pharmaceutical medications are identified and quantitatively analyzed using a variety of techniques, including spectrophotometry, electroanalytical techniques (primarily voltammetry), titrimetric, fluorimetry, and chromatographic procedures such as TLC, GC, HPLC, and capillary electrophoresis (CE) [6–8].

Triclabendazole (TCB), 6-chloro-5-(2,3 dichlorophenoxy)-2-methyl thio-benzimidazole), is a common anthelmintic used to treat and prevent liver fluke infection in ruminants caused by *Fasciola hepatica* and *F. gigantica* [9]. These types of flukes are widespread and result in large financial losses for the cattle sector. Because BZD binds to the β-tubulin colchicine-binding site and prevents it from polymerizing into microtubules, it has nematicidal activity. Although TCB exhibits strong efficacy against both the mature and an early juvenile stage of liver fluke in cattle, goats, and sheep, many anthelmintic medications are only effective against the mature form of the parasite [10]. The maximum residue limits (MRLs) for TCB in the muscle, liver, fat, skin, and kidney of cattle and sheep are defined by the WHO and range from 100 to 850 μg/kg [11]. Additionally, MRLs for the muscle, fat, liver, kidney, and milk from all ruminants, ranging from 10 to 250 μg/kg, have been proposed by the European Union [9]. The total extractable residues that can be converted to keto-TCB through oxidation is known as the residual TCB for ruminants. It is obvious that precise and reliable analytical techniques are needed to monitor residual TCB and its metabolites in cattle products such as milk and meat.

Ivermectin (IVM) is a potent semisynthetic antiparasitic drug that is widely used to treat parasites in veterinary medicine. IVM is also used for humans suffering with lymphatic filariasis and onchocerciasis, also known as river blindness [12]. IVM is a member of the naturally occurring avermectin class, which is produced by the soil-dwelling actinomycete *Streptomyces avermitilis.* IVM is highly efficient in the treatment of various ectoparasites such as mites, lice, ticks, and parasitic fly larvae. The binding of AVM to the GABA receptor in the mammalian host's central nervous system is linked to their toxicity. The reported LD-50 values of IVM are as follows: 28–30 mg/kg for oral and i/p administration in mice; 80 mg/kg for oral administration in dogs; 40 mg/kg for cutaneous administration in rabbits; and more than 200 mg/kg for oral treatment in monkeys. Livestock only partially metabolizes this medicine; large amounts of the parent drug are expelled primarily in the form of feces, which contaminate the aquatic environment. Therefore, it is necessary to develop highly efficient analytical method for the determination and quantification of IVM in animals products and water.

Various analytical methods have been reported for the determination of TCB and IVM separately and its combination dosage form including HPLC methods [13], LC–MS methods [14–16], CE [17, 18], spectrofluorimetric method [19], and spectrophotometric method [19]. Most of these methods have analyzed either TCB or IVM individually or with some other drugs. It is very challenging to analyze a specific drug in the mixture in which one drug is in very low dosage form. The mixture analyzed in the current study composed of TCB (12%) and IVM (0.2%) while the remaining 87.8% are excipients in the suspension, so it was very difficult to assess both when one drug is present in such a low dosage form. The massive variances arisen in the peak heights due to the concentration of TCB and IVM in the suspension. Similarly, development of an isocratic HPLC method has several advantages over the gradient HPLC method such as cost-effectiveness and rapidness and can be conducted on a simple HPLC system as there is only one mobile phase consumed throughout the analysis. Moreover, ICH parameters such as system appropriateness, linearity, precision, accuracy, the limit of detection (LOD) and limit of quantification (LOQ) should be validated for a new method to confirm its suitability and use for routine analysis of targeted analytes.

In the current study, a simple RP-HPLC method was developed for the simultaneous determination of TCB and IVM in drug suspensions. The mixture was separated under isocratic elution conditions using a C18 column (4.6 mm × 250 mm, 5 μm); mobile phase acetonitrile/water 50/50 (v/v). The developed method is very sensitive with very low detection limits (LOD) of 0.058 mg/mL and 0.178 mg/mL and LOQ of 0.178 μg/mL and 0.340 μg/mL for TCB and IVM, respectively, with relative standard deviation (RSD) < 2%. The developed method is accurate, fast, precise, and cost-effective, for estimating TCB and IVM simultaneously in pharmaceutical suspension dosage form using HPLC-UV under isocratic elution conditions.

## 2. Materials and Methods

### 2.1. Chemicals and Materials

The reference standard of TCB and IVM and as a dosage form Endoplus 12.2% oral suspension 100 mL (each ml containing TCB 120 mg and IVM 2 mg) were supplied by Hawk BioPharma, Rawat, Rawalpindi, Pakistan. HPLC grade acetonitrile (Sigma-Aldrich), methanol (Sigma-Aldrich), water, nylon membrane filters of pore size 0.45 micron, and syringe filters having a diameter of 13 mm and pore size of 0.22 μm were purchased from SciTech, Pakistan.

### 2.2. Instrumentation

Wufeng HPLC equipped with a single LC100 pump, LC100 UV detector, manual injection port, and 20-μL glass syringe was used. The rest of the apparatuses used were a weighing balance TS214S model (Sartorius; made in Germany), ultrasonic bath DSA100-SK1 ROHS, filtration assembly, and hotplate and stirrer MS300HS.

### 2.3. Sample Preparation

TCB (100 mg) and IVM (5 mg) were added to a 250-mL volumetric flask containing 50 mL of methanol followed by stirring and sonication for 10 min for homogenization. Then, 5 mL form this mixture was subsequently added into a 50-mL volumetric flask and further diluted using methanol and filtered using syringe filters with 0.45-μm pore sizes [20]. The stock solutions were diluted to different concentrations for chromatographic analysis.

### 2.4. HPLC Analysis

An HPLC system, Wufeng HPLC, equipped with a single LC100 pump, LC100 UV detector, manual injection port, 20-μL glass syringe, and C18 column (Welchrom) (4.6 × 250 mm, 5 μm) was used for analysis. The separation was carried out at an ambient temperature with a flow rate of 1.5 mL/min and the detection wavelength of 254 nm. An equal volume of standard and sample solutions was injected separately, and the chromatograms were recorded. Peak areas were measured after the standard preparation performed for five runs of HPLC analysis. Five replicates were measured, and % RS was less than 2.0%. The peak area of the chromatograms obtained from the standard and sample solution was used to infer the results. The percentage of drug in the sample was calculated using equation (1) [21].

(1) $$\begin{array}{c} \text{Assay }\left(\%\right)=\frac{\text{Peak area of the sample}}{\text{Peak area of standard}}. \end{array}$$

### 2.5. Method Validation

RP-HPLC method validation for pharmaceutical analysis depends on a number of critical factors, such as robustness, range, linearity, accuracy, and precision. In the current study, the developed RP-HPLC method was validated according to the international committee for harmonization of technical standards for medicinal products for human use (ICH). Validation characteristics such as selectivity, system suitability, linearity, sensitivity, precision, accuracy, ruggedness, and robustness were evaluated [22, 23].

#### 2.5.1. Selectivity

The capability of an analytical method to precisely identify and measure the target analyte in a complicated sample matrix while keeping it apart from any possible interfering substances is known as selectivity. The selectivity of HPLC method is crucial for pharmaceutical analysis since samples may include different excipients, degradation products, or contaminants. In the current study, RP-HPLC method's selectivity was evaluated by examining the placebo samples to make sure that there are no interfering peaks at the analyte's retention time. Furthermore, the selectivity of the RP-HPLC method was examined by comparing the chromatograms obtained with the peak purity index (PPI) values of the analytes. The best possible analytical conditions were used to analyze the samples [24, 25].

#### 2.5.2. System Suitability

System suitability testing is a crucial part of the RP-HPLC method validation, especially in pharmaceutical analysis. It involves a set of tests and criteria to ensure that the chromatographic system is suitable for the intended analysis. Various parameters such as resolution, retention time, peak symmetry, selectivity or specificity, linearity, accuracy and precision, and robustness are among the parameters that are typically evaluated to assess the system's suitability. All these parameters were evaluated for TCB and IVM analysis which are described below.

#### 2.5.3. Linearity

Solutions containing various concentrations of TCB and IVM (5.00, 10.0, 25.0, 50.0, and 100 μg/mL) were analyzed in order to conduct the linearity of the developed RP-HPLC method. Using least-squares linear regression analysis, the peak areas of TCB and IVM were plotted against their concentrations to construct the calibration plots. To achieve peak area repeatability, the samples were injected three times. Ultimately, the coefficient correlation of each concentration as well as the average of all the peak regions of the concentrations was computed.

#### 2.5.4. Sensitivity

Sensitivity of the developed RP-HPLC method for TCB and IVM analyses was checked through the LOD and LOQ values based on signal-to-noise ratios of 3:1 and 10:1, respectively.

##### 2.5.4.1. LOD

The lowest concentration of an analyte in a sample at which it can be identified is known as the LOD. By multiplying the standard deviation by factor 3.3 and dividing the result by the curve's slope, the LOD was calculated [26]. The LOQ was determined by constructing a linear regression curve with relatively low concentrations of the target chemical. Equation (2) was utilized to compute the LOD based on the regression lines. Samples containing an analyte in the range of the LOD were subjected to study specific calibration curve. The standard deviation of y-intercepts of regression lines was used as the standard deviation.

(2) $$\begin{array}{c} \text{LOD}=\frac{\left(3.3\;x\;\sigma \right)}{S}, \end{array}$$

where “*σ*” is standard deviation of the intercepts and “*S*”*”* is average slope of the calibration curve.

##### 2.5.4.2. LOQ

LOQ is the minimum analyte concentration in any sample that can be quantified with accuracy and precision. The LOQ is determined by multiplying standard deviation with factor 10 and dividing by the slope of the curve. It was calculated by using the following formula. The LOQ was calculated by using equation (3) [26].

(3) $$\begin{array}{c} \text{LOQ}=\frac{10\;x\;\sigma }{S}, \end{array}$$

where “*σ*” is standard deviation of the intercepts and “*S*” is average slope of the calibration curve [27].

#### 2.5.5. Recovery

Three replicates of TCB and IVM at four distinct concentration levels (0.15, 5.0, 25.0, and 100.0 μg/mL) were run on the same day and three consecutive days for inter- and intraday precision and accuracy. At each concentration level, the %RSD and relative error (RE) were determined for accuracy and precision, respectively. After injecting samples in triplicate at each of the three doses, the average peak areas were determined. By calculating the % recovery of the analytes over the assay ranges, the accuracy was determined as shown in equation (4).

(4) $$\begin{array}{c} \%\text{ Recovery}=\frac{A\left(\text{fr}\right)\times V\left(\text{fr}\right)\times V\left(\text{inj}\right)\times 100}{A\left(\text{crude}\right)\times V\left(\text{crude}\right)\times V\left(\text{Injfrac}\right)\times \text{Df}}, \end{array}$$

where Afr and Vfr are the peak area and volume of the collected fraction, V (inj) is the injection volume, A (crude) is the peak area of the crude solution, V(crude) is the crude volume, V (injfrac) is the injection volume of the fraction, and Df is the dilution factor of the crude sample solution for injection.

#### 2.5.6. Precision and Accuracy

Six sample injections were run, and the resultant chromatogram was used to calculate the peak area of six replicates. The % RSD, standard deviation, and mean value were calculated. For TCB and IVM, the % RSD of six replicates at 100% of the test concentration was less than 2%.

#### 2.5.7. Ruggedness

The laboratory variation parameters of intraday variation and distinct analyst variation were chosen for the computation of intermediate precision or ruggedness. To observe day-to-day variance in the outcome of the suggested analytical approach, different days were chosen and samples were injected in triplicates. In order to verify for variations in the results of different analysts, two analysts were chosen, and they were then required to prepare samples and run them through an HPLC.

#### 2.5.8. Robustness

While the analytical procedure was being developed, the robustness parameter was ascertained. The procedure was tested with varying flow rates and mobile phase compositions to ensure its robustness. The samples were injected in triplicate at 1 mL/min, 1.5 mL/min, and 2.0 mL/min to test the flow rate change. Samples were injected in triplicates into an HPLC system that was saturated with various mobile phase concentrations in order to affect a shift in the mobile phase. The mobile phase compositions that were chosen were as follows: ACN: water 50:50 (v/v), 60:40 (v/v), and 70:30 (v/v).

### 2.6. Statistical Analysis

Each experiment was repeated three times. The results were expressed as regression lines or correlation coefficients, and standard mean deviations and RSDs. Based on the regression lines, LOQ and LOD were calculated. Origin Pro and Microsoft Excel were used for the regression analysis.

## 3. Results and Discussion

### 3.1. Method Development and Optimization

**Figure 1:**
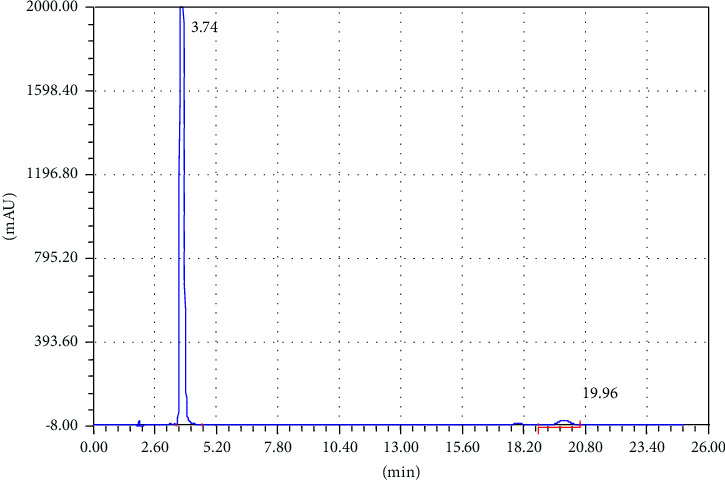
Chromatogram of standard mixture of TCB (tR = 3.74 min) and IVM (tR = 19.96 min). Elution conditions: mobile phase ACN/water (50:50, V/V) and flow rate 1.5 mL/min.

**Figure 2:**
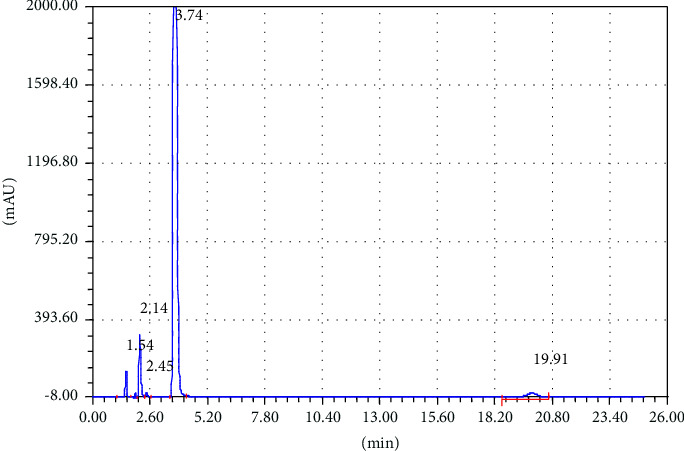
Chromatogram of suspension of TCB (tR = 3.74 min) and IVM (tR = 19.91 min). Elution conditions: mobile phase ACN/water (50:50, V/V) and flow rate 1.5 mL/min.

To achieve good separation of the targeted analytes, different parameters were tested such as appropriate column, mobile phase composition with different proportions, wavelength, and column temperature. Various mobile phases including different organic modifiers such as methanol, 2-propanol, and acetonitrile (ACN) with water at different proportions were tried for the separation of TCB and IVM. Among these mobile phases, the mixture of ACN/water shows excellent separation for the targeted analytes; therefore, ACN/water was selected as the mobile phase for further experiments. The separation of TCB and IVM was carried out using 50/50 ACN/water (v/v) at the flow rate of 1.5 mL/min and UV detection wavelength of 254 nm. The effect of mobile phase flow rate was on the separation of TCB and IWM was checked using different flow rates (1.0 mL/min, 1.5 mL/min, and 2.0 mL/min), and it was observed that a flow rate of 1.5 mL/min gave appropriate separations with reasonable run time. The chromatograms of reference standard and sample of TCB and IVM are shown in Figures 1 and 2, respectively. The chromatogram in Figure 1 shows that the retention times of TCB and IVM are 3.74 min and 19.96 min, respectively. The retention time of TCB is less than IVM because TCB is highly polar drug and does not interact strongly with reversed-phase C18 column and thus eluted early. On the other hand, IVM has a highly conjugated aromatic ring which interacts with the nonpolar stationary phase (C18) by hydrophobic interactions. Similarly, the chromatogram in Figure 1 shows only two peaks while the chromatogram in Figure 2 shows several small peaks which look like the impurities or some degradation products. Most probably, these small peaks may be some impurities eluted from the column or injector because the intensities of these peaks are very low as compared to the major peak representing the targeted analytes. Such peaks are common in chromatography owing to the column overloading or insufficient washing of chromatographic columns or injectors. Owing to small intensities, such peaks could be neglected because these peaks do not affect the method's specificity.

### 3.2. Method Validation

#### 3.2.1. System Suitability

System suitability parameters were evaluated using six different replicates of the same concentration of the reference standard mixture and suspension of TCB and IVM both containing 3 mg/L of TCB and 0.005 mg/L of IVM. Peak areas of the six homogenous sample replicates were measured, and the values of various parameters such as mean, standard deviation, and % RSD were measured. The % RSD values of the six replicates were less than 2%, which is the acceptance criterion of RSD < 2.0, which shows that the developed method was suitable for analysis. Moreover, retention time, tailing factor, peak resolution, theoretical plates, and repeatability were assessed. The result of the system appropriateness study of the developed method is shown in Table 1.

#### 3.2.2. Linearity

**Figure 3:**
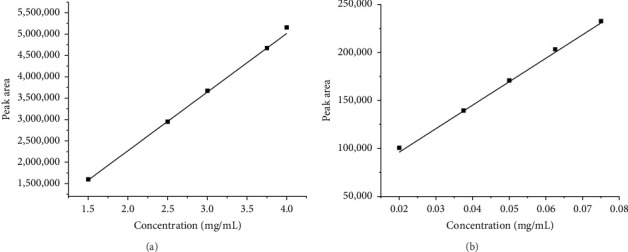
Linearity of the developed method for TCB (a) and IVM (b).

During method validation, the linearity of the method was evaluated by using five different replicates of the solution with different concentrations ranging from 50% to 150%. The method was found to be linear, and results are shown in Table 2. The linearity graphs (conc vs. peak area) of TCB and IVM are shown in Figures 3(a) and 3(b), respectively, with correlation coefficient of *R*^2^ values of 0.999, which show the linearity of the developed method for both TCB and IVM. It was found that the current method's linearity and range fell within an acceptable limit, i.e., 0.999. The numerical values of linearity of TCB and IVM are given in Table 2. The results presented in Table 2 show that the peak areas of both drugs, TCB and IVM, increased with increasing concentration of these drugs. The results provided in Table 2 are in close agreement with Figure 3.

#### 3.2.3. Accuracy and Recovery

Accuracy and recovery of TCB and IVM were determined at concentration levels 50%, 100%, and 150%. The mean recovery values for TCB and IVM were found to be 100.87% and 99.83% with standard deviation of 0.814 and 1.424, respectively, as shown in Table 3.

### 3.3. Precision of the Developed Method

#### 3.3.1. Repeatability

Repeatability is the precision of the analytical method when repeated by the same analyst under the same working conditions using the same equipment, reagents, and operation conditions over a short interval of time. The repeatability of the developed method was assessed by evaluating six replicates of the TCB and IVM analysis (both standard and sample suspension), and it was observed that the % RSD of method repeatability was 0.22% and 0.24% for bulk drugs and assays, respectively. Various parameters of method precision such as % assay, mean assay (%), standard deviation, and % RSD of TCB and IVM are given in Table 4.

#### 3.3.2. Intermediate Precision (Ruggedness)

Ruggedness or intermediate precision describes the general reproducibility of results when an analysis is carried out under a range of circumstances. It evaluates the effects of several variables, including ambient circumstances, instrumentation, and analyst differences, on the outcomes of a certain analytical method. For the analytical procedure to yield dependable and consistent results in pharmaceutical analysis, even under slight variations in the experimental conditions, intermediate precision is essential. An analytical method's ability to exhibit intermediate precision or ruggedness offers confidence that the procedure can consistently produce correct and trustworthy results under a variety of conditions. This is crucial for quality assurance and regulatory compliance in fields where accurate measurements are vital. Table 4 provides a number of intermediate precision measurements, including percentage assay, mean assay (%), standard deviation, and percentage RSD of TCB (Table 4[a]) and IVM (Table 4[b]). The results show that the mean assay (%) of TCB and IVM is 100.05% and 100.09%, respectively, as shown in Table 4(a) and 4(b), respectively. Similarly, the %RSD values of TCB and IVM are 0.224 and 0.242, respectively, which show that the developed method is highly precise and reproducible (Table 4).

#### 3.3.3. Robustness of the Developed Method

An analytical method is said to be robust if it can withstand minor changes in the experimental settings and method parameters, such as pH, temperature, composition of the mobile phase, and flow rate. Small variations in these variables should not affect the consistency and dependability of the outcomes produced by a robust approach. Conducting tests in which one parameter is purposefully changed within a certain range at a time while maintaining other conditions constant is necessary to evaluate the robustness of the procedure. After then, the data are examined to see if the differences have any appreciable effect on the method's accuracy or performance. An analytical method's robustness is crucial to ensuring that it can tolerate typical changes without sacrificing the caliber of the results. The method's robustness was assessed by deliberately making small changes in the flow rate, and wavelength results are shown in Table 5(a) and 5(b).

#### 3.3.4. LOD

The lowest concentration of an analyte in a sample that is readily detectable but not always quantifiable is known as the LOD. LOD can be calculated by multiplying the standard deviation with 3.3 and dividing the result by the curve's slope. At a signal-to-noise ratio (S/N) of 3, LOD was measured for TCB and IVM. Through three injections of each analyte at doses ranging from 50% to 150%, the LOD was experimentally confirmed.  Table S1 displays the computed LODs for TCB and IVM, which are 0.058 mg/mL and 0.112 μg/mL, respectively.

#### 3.3.5. LOQ

The lowest concentration of an analyte in a sample that can be precisely and reliably quantified is known as the LOQ. The standard deviation is multiplied by ten to get the LOQ, which is then divided by the curve's slope. The *y*-intercept standard deviation of the regression line was utilized as the standard deviation. A threshold for quantification was set at a signal-to-noise ratio (S/N) of 9. LOQ for TCB and IVM was experimentally confirmed with three injections of each medication at 50%–150% doses.  Table S2 shows the LOQ values of TCB and IVM which are 0.178 μg/mL and 0.340 μg/mL, respectively.

### 3.4. Specificity

**Figure 4:**
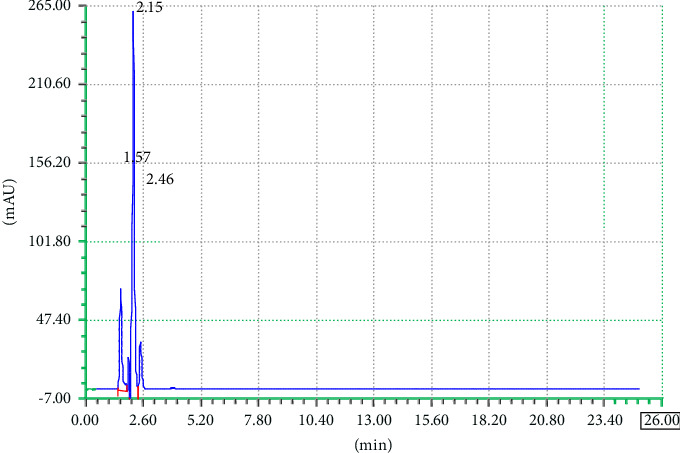
Chromatogram of the specificity placebo interference.

**Figure 5:**
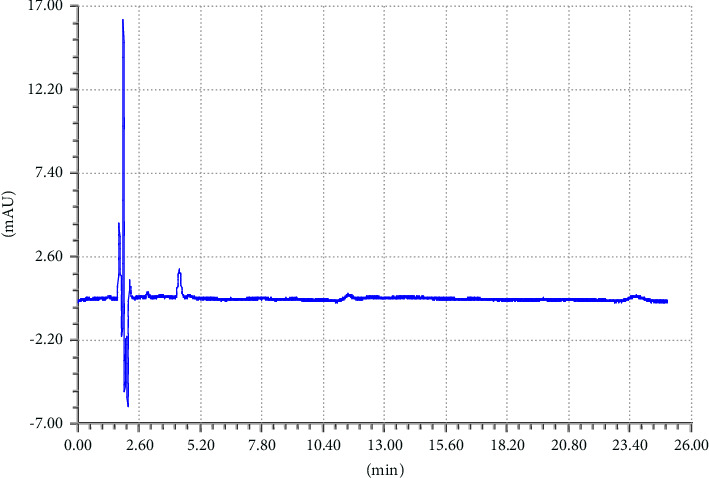
Chromatogram of the specificity blank interference diluent.

**Figure 6:**
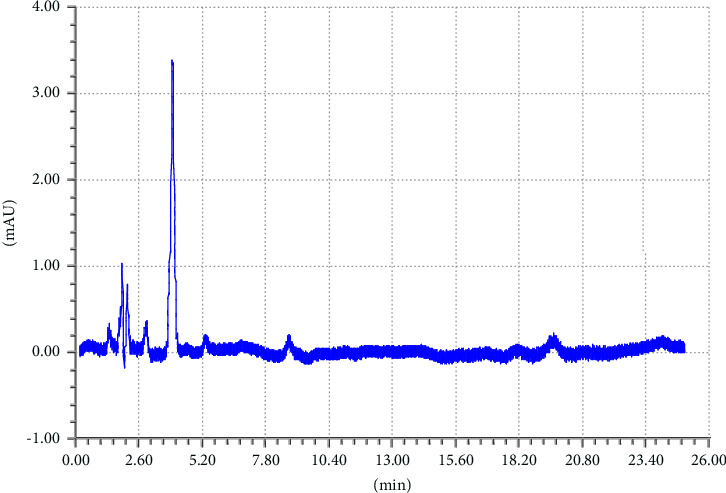
Chromatogram of the specificity blank interference mobile phase.

An analytical method's specificity is its capacity to assess the target analyte with accuracy in the presence of other analytes or interferences. It is necessary to make sure that the other analytes in the sample would not interfere with the target analyte. Validation tests are commonly carried out to evaluate specificity by ascertaining whether the accuracy and precision of the method are impacted by any possible interferences from matrix components, contaminants, or closely related compounds. To prove there is no interference, this may entail performing selectivity tests or evaluating samples containing known interfering substances. The objective of specificity is to verify that other analytes are not interfering and that the method's response is solely caused by the presence of the target analyte. The method specificity was checked by comparing the sample chromatograms, standard, and the corresponding placebo as shown in chromatograms Figures 4, 5, and 6. The chromatogram in Figure 4 shows that there is no peak at the retention times of TCB and IVM when HPLC run of the blank placebo, interference, and mobile phase were recorded. The retention times of TCB and IVM under these elution conditions are 3.7 min and 19.9 min, as shown in Figure 1.

Similarly, the blank interference diluent was carried out to check if there is any peak in the retention time slots of targeted drugs TCB and IVM. The same elution conditions were used for the chromatographic run. A specific quantity of diluent (methanol) was injected, and the chromatogram was recorded as shown in Figure 5. The chromatogram in Figure 5 shows that there is no prominent peak at the retention time slots of TCB and IVM.

Similarly, the blank interference diluent was carried out to check if there is any peak in the retention time slots of targeted drugs TCB and IVM. The same elution conditions were used for the chromatographic run. A specific quantity of the mobile phase was injected, and the chromatogram was recorded, as shown in Figure 6. The chromatogram in Figure 6 shows that there is no prominent peak at the retention time slots of TCB and IVM.

## 4. Conclusion

A RP-HPLC method was developed for the analysis of TCB and IVM in suspension dosage form using an isocratic elution condition. The method developed for the analysis of TCB and IVM is linear with a correlation coefficient of 0.999. The intraday precision of TCB and IVM was found to be 100.98% and 100.02% with RSD of 0.18% and 0.24%, respectively. The established RP-HPLC method is reproducible and satisfies the formal specifications for international protocols. The developed method may be applied to the identification and measurement of TCB and IVM in a variety of samples such as pharmaceutical dosage forms and suspensions. Furthermore, the developed RP-HPLC method is simple, accurate, linear, and robust as compared to other reported methods. In the future, the established RP-HPLC method will be tested for the analysis of other comparable drugs in tablet and suspension dosage forms.

## Figures and Tables

**Table 1:** System suitability parameters for the established RP-HPLC method for the analysis of triclabendazole and ivermectin.

Parameters	Triclabendazole	Ivermectin
Retention time (Rt)	3.73 ± 0.349%	19.83 ± 0.453
Theoretical plates (N)	2109 ± 1.200	3394 ± 1.96
Tailing factor (Tf)	1.73 ± 1.51%	0.82 ± 1.67%
Resolution (Rs)	—	17.55 ± 1.87%
Repeatability	3540296 ± 0.784%	163,576 ± 0.389%

**Table 2:** Linearity of triclabendazole and ivermectin.

TCB			IVM		
Linearity level	Concentration (mg/mL)	Peak area	Linearity level	Concentration (mg/mL)	Peak area
50	1.5	1601118.5	1	0.02	100813.1
75	2.5	2949947.8	2	0.0375	139528.5
100	3	3673425.3	3	0.05	170876.1
125	3.75	4673558.3	4	0.0625	203361.3
150	4	5154921	5	0.075	232598.9

**Table 3:** Recovery of TCB and IVM using the RP-HPLC method for analysis.

Drug	Peak area (%)	Concentration (mg/mL)	Standard	Essay	%Peak area	Recovery peak area
Triclabendazole	50	1.5	1811118.5	3557462.9	50.91	101.821
	100	3	3573425.3	3557462.9	100.45	100.449
	150	4	5354921.0	3557462.9	150.53	100.351

Ivermectin	50	0.025	81813.1	161459.1	50.671	101.342
	100	0.05	160876.1	161459.1	99.639	99.639
	150	0.075	238598.9	161459.1	147.777	98.518

**Table 4:** Repeatability of precision of TCB and IVM.

	Set no.	Assay (%)	Mean assay (%)	Std dev	RSD (%)
*(a) Triclabendazole*					
Method precision	1	101.32	100.05%	0.224	0.221
	2	101.27			
	3	100.94			
	4	100.89			
	5	101.14			
	6	100.76			

Intermediate precision	1	100.95	100.98%	0.183	0.181
	2	101.13			
	3	101.22			
	4	100.73			
	5	101.05			
	6	100.84			

*(b) Ivermectin*					
Method precision	1	100.51	100.09	0.242	0.242
	2	99.96			
	3	99.88			
	4	100.23			
	5	100.06			
	6	99.90			

Intermediate precision	1	99.82	100.02	0.377	0.377
	2	99.75			
	3	100.16			
	4	99.76			
	5	100.73			
	6	99.93			

**Table 5:** Robustness data effect of change in flow rate (a) and wavelength (b) of TCB and IVM.

	Flow rate (mL/min)	Peak area	Assay (%)	Retention time	Theoretical plates	Tailing factor
*(a) Effect of change in flow rate*						
Triclabendazole	1.400	3559543	100.05	3.68	2112	1.76
	1.500	3557872	100.01	3.74	2137	1.74
	1.600	3556164	99.96	3.87	2145	1.75

Ivermectin	1.400	161539	100.04	19.56	3386	0.83
	1.500	161511	100.03	19.75	3400	0.83
	1.600	161508	100.03	20.01	3411	0.84

	**Wavelength (nm)**	**Peak area**	**Assay (%)**	**Retention time**	**Theoretical plates**	**Tailing factor**

*(b) Effect of change in wavelength*						
Triclabendazole	252	3558614	100.03	3.71	2124	1.76
	254	3557824	100.01	3.72	2158	1.73
	256	3558991	100.04	3.76	2112	1.74

Ivermectin	252	161533	100.04	19.34	3413	0.85
	254	161555	100.05	19.67	3399	0.84
	256	161609	100.09	19.70	3427	0.82

## Data Availability

The data are provided within the manuscript while additional data are provided in the Supporting information file.

## Supporting Information

Table S1: Limit of detection (LOD) and limit of quantification (LOQ) of TCB.

Table S2: Limit of detection (LOD) and limit of quantification (LOQ) of IVM.
