# Supplementary material for: Development of RP-HPLC Method for Simultaneous Determination of Triclabendazole and Ivermectin in Pharmaceutical Suspension Dosage Form
Source: J Anal Methods Chem. 2025 Apr 22;2025:5522915. doi: 10.1155/jamc/5522915 (PMC12041623; doi:10.1155/jamc/5522915)
Supplement: Supporting Information — Additional supporting information can be found online in the Supporting Information section. [file 5522915.f1.docx]

**<Supplementary information>**

**Development of RP-HPLC method for simultaneous determination of** **triclabendazole and ivermectin in pharmaceutical suspension dosage form**

Taj Ur Rahman^1^, Ajmal Zaman^2^, Ali Bahadur^3^, Muhammad Aurang Zeb^1^, Wajiha Liaqat^1^, Eman Y. Santali^4^, Sarah Alharthi^5,6^, Ruwida M K Omar^7^, Saif A Alharthy^8,9^ and Ashraf Ali^10*^

*^1^*Department of Chemistry, Mohi-Ud-Din Islamic University, Nerian Sharif, Azad Jammu & Kashmir.

^2^*Department of Transdisciplinary Studies, Graduate School of Convergence Science and Technology, Seoul National University, Seoul, 08826, South Korea.*

*^3^Department of Chemistry, School of Natural Sciences (SNS), National University of Science and Technology (NUST), H-12, Islamabad, 46000, Pakistan.*

*^4^Department of Pharmaceutical Chemistry, College of Pharmacy, Taif University, Taif 21944, Saudi Arabia*

^5^Department of Chemistry, College of Science, Taif University, P.O. Box 11099, Taif 21944, Saudi Arabia.

^6^Research Center of Basic Sciences, Engineering and High Altitude, Taif University, Taif 21944, Saudi Arabia.

*^7^Pharmaceutical Chemistry Department, Faculty of Pharmacy. University of Benghazi, Libya*

*^8^Department of Medical Laboratory Sciences, Faculty of Applied Medical Sciences, King Abdulaziz University,* P.O. Box 80216, Jaddah 21589, Saudi Arabia.

*^9^*King Fahad Medical Research Center, *King Abdulaziz University,* P.O. Box 80216, Jaddah 21589, Saudi Arabia.

*^10^Department of Chemistry, Faculty of Physical & Applied Sciences, The University of Haripur, Haripur 22620, Pakistan*

**Correspondence:* [*ashrafaliswati@gmail.com*](mailto:ashrafaliswati@gmail.com) *(Dr. Ashraf Ali)*

**Table S1.** Limit of detection (LOD) of TCB and IVM

| Sample | Conc. % | LOD of TCB | LOD of IVM |
| --- | --- | --- | --- |
| 1 | 80 | 0.058 mg/ml | 0.112 µg/ml |
| 2 | 90 |  |  |
| 3 | 100 |  |  |
| 4 | 110 |  |  |
| 5 | 120 |  |  |

Table S2. Limit of quantification (LOQ) of TCB and IVM.

| Sample | Conc. % | LOQ of TCB | LOQ of IVM |
| --- | --- | --- | --- |
| 1 | 80 | 0.178 µg/ml and | 0.34 µg/mL |
| 2 | 90 |  |  |
| 3 | 100 |  |  |
| 4 | 110 |  |  |
| 5 | 120 |  |  |
